# Carriage of antimicrobial-resistant bacteria in a high-density informal settlement in Kenya is associated with environmental risk-factors

**DOI:** 10.1186/s13756-021-00886-y

**Published:** 2021-01-22

**Authors:** Sylvia Omulo, Eric T. Lofgren, Svetlana Lockwood, Samuel M. Thumbi, Godfrey Bigogo, Alice Ouma, Jennifer R. Verani, Bonventure Juma, M. Kariuki Njenga, Samuel Kariuki, Terry F. McElwain, Guy H. Palmer, Douglas R. Call

**Affiliations:** 1 Paul G. Allen School for Global Animal Health, Washington State University, Pullman, WA USA; 2 Washington State University Global Health-Kenya, Nairobi, Kenya; 3 Center for Global Health Research, Kenya Medical Research Institute, Kisumu, Kenya; 4 Centers for Disease Control and Prevention, Nairobi, Kenya; 5 Center for Microbiology Research, Kenya Medical Research Institute, Nairobi, Kenya; 6 Nelson Mandela African Institution for Science and Technology, Arusha, Tanzania

**Keywords:** Sanitation, Antimicrobial resistance, Informal settlement, *E. coli*, Kenya

## Abstract

**Background:**

The relationship between antibiotic use and antimicrobial resistance varies with cultural, socio-economic, and environmental factors. We examined these relationships in Kibera, an informal settlement in Nairobi—Kenya, characterized by high population density, high burden of respiratory disease and diarrhea.

**Methods:**

Two-hundred households were enrolled in a 5-month longitudinal study. One adult (≥ 18 years) and one child (≤ 5 years) participated per household. Biweekly interviews (n = 1516) that included questions on water, sanitation, hygiene, and antibiotic use in the previous two weeks were conducted, and 2341 stool, 2843 hand swabs and 1490 drinking water samples collected. Presumptive *E. coli* (n = 34,042) were isolated and tested for susceptibility to nine antibiotics.

**Results:**

Eighty percent of presumptive *E. coli* were resistant to ≥ 3 antibiotic classes. Stool isolates were resistant to trimethoprim (mean: 81%), sulfamethoxazole (80%), ampicillin (68%), streptomycin (60%) and tetracycline (55%). Ninety-seven households reported using an antibiotic in at least one visit over the study period for a total of 144 episodes and 190 antibiotic doses. Enrolled children had five times the number of episodes reported by enrolled adults (96 vs. 19). Multivariable linear mixed-effects models indicated that children eating soil from the household yard and the presence of informal hand-washing stations were associated with increased numbers of antimicrobial-resistant bacteria (counts increasing by 0·27–0·80 log_10_ and 0·22–0·51 log_10_ respectively, depending on the antibiotic tested). Rainy conditions were associated with reduced carriage of antimicrobial-resistant bacteria (1·19 to 3·26 log_10_ depending on the antibiotic tested).

**Conclusions:**

Antibiotic use provided little explanatory power for the prevalence of antimicrobial resistance. Transmission of resistant bacteria in this setting through unsanitary living conditions likely overwhelms incremental changes in antibiotic use. Under such circumstances, sanitation, hygiene, and disease transmission are the limiting factors for reducing the prevalence of resistant bacteria.

## Introduction

Numerous organizations have called attention to the increasing prevalence of antimicrobial resistance (AMR) worldwide [1]. Efforts to preserve the efficacy of existing antibiotics have focused predominantly, if not exclusively, on improving prescription guidelines and compliance to reduce unnecessary antibiotic use. This “stewardship focus” is led by high-income countries in North America, Europe, and Oceania where robust healthcare infrastructure and regulatory frameworks are present. Nevertheless, these countries comprise only 15% of the world’s population and are poorly representative of the global burden of infectious disease [2–4].

In 2013 the United Nations Human Settlements Program estimated that a quarter of the world’s urban population lived in slums [5]; informal settlements characterized by high population density, poor sanitation and lack of consistent access to clean water. These characteristics promote the spread of infectious diseases and the demand for antibiotics, when available [6–8], and support the observations by Collignon et al. [9] that countries with poor infrastructure, poor governance and limited public health expenditures have a higher prevalence of antimicrobial-resistant bacteria compared with countries that rank better by these metrics. Consequently, while encouraging antibiotic stewardship might be important for limiting AMR, it may not be an effective strategy for communities where exposure to unhygienic conditions and rapid spread of pathogens overwhelm individual decisions regarding antibiotic use, whether within or outside the healthcare system.

We assessed the relationship between sanitation, antibiotic use, and antimicrobial resistance in Kibera. We surmise that socio-economic and environmental determinants likely impact both antibiotic use and antimicrobial resistance in Kibera and similar impoverished communities.

## Methods

### Study area

This study involved two villages in Kibera—Soweto and Gatwekera. Kibera, an informal settlement in Nairobi—Kenya, is one of the largest slums in Africa [5]. Residents of Kibera suffer a high incidence of infectious disease, including diarrhea, which is compounded by a lack of safe and hygienic sanitation facilities [3, 10]. Correspondingly, antibiotic use is common; a 2016 survey showed that 87% of Kibera respondents reported having used an antibiotic in the 12-month period preceding the survey [11]. Soweto and Gatwekera have a population density of 55,000–84,000 persons/km^2^ [12] and are part of a population-based infectious disease surveillance (PBIDS) program. This program collects household and clinic data on common infectious disease syndromes for an enumerated population of ~ 25,000 individuals and is operated and supported jointly by the Kenya Medical Research Institute—KEMRI, Carolina for Kibera, and the U.S. Centers for Disease Control and Prevention—CDC [3].

### Study design

#### Household selection

Two-hundred households were randomly selected from a census of 5320 households that participate in PBIDS. Selection was restricted to households with at least one adult (≥ 18 years) and one child (≤ 5 years), and to one household per housing block. This strategy maximized variation in our sample; adults and children vary in their antibiotic use and sanitation practices while household locations determine access to antibiotic sources and sanitation facilities. Within each household, two members were invited to enroll into the study; an adult with knowledge of household sanitation and healthcare practices, and the youngest of children aged ≤ 5 years. Residents who were routinely engaged in daytime activities (e.g., work or school) outside the household were excluded from enrollment to minimize loss to follow-up.

#### Survey data collection

Households were visited once every two weeks, for a total of nine study visits, between August 2015 and January 2016. This period encompassed a dry (August–October) and wet (November–January) season. Local monthly average rainfall (mm) was retrieved from the v7 Tropical Rainfall Measuring Mission Multi-Satellite Precipitation Analysis algorithm [13]. At each visit, data on self-reported sanitation- (Additional file 1) and antibiotic use practices (Additional file 2) during the two weeks preceding each visit were recorded. The sanitation survey tool was modified from a pre-tested questionnaire [14] that expanded upon a Joint Monitoring Programme tool for Water Supply and Sanitation [15]. The antibiotic use survey tool was developed and piloted in select households within Kibera (excluded from the main study). This tool addressed antibiotic types and sources, reasons for antibiotic use, dose completion and duration of use. During each visit, household respondents were reminded to retain used medication packages for subsequent data abstraction. Observational data for household drinking water storage containers, toilet types and hand-washing stations were collected at enrollment, and at visits 8 and 9.

#### Sample collection

Up to five samples were collected from each household at each visit, including a stool sample and a hand-swab from the two enrolled respondents, and a water sample from the household’s drinking water reserve. Households were trained on acceptable stool collection and packaging methods during the enrollment visit. Thereafter, a stool collection kit was supplied on the eve of each follow-up visit to enable participants to collect fresh stool on the day of the visit. Hand swabs and water samples were collected by field officers on the day of the visit. To collect a hand-swab, a sterile polyester-tipped applicator (Puritan, Guilford, ME) was moistened in sterile phosphate-buffered saline (PBS) and rolled over the palm of a participant’s right hand, in between the fingers and under the nails. The swab was then immersed into a vial containing 1 mL of sterile PBS, capped and labeled. Water samples were collected by asking the adult respondent to provide a cup of drinking water, which was subsequently transferred into two sterile 50 mL tubes. All samples were maintained in iceboxes until transported to a KEMRI laboratory located within Kibera for processing within 6 h of collection.

#### Sample processing methods

Stool samples were processed by adding 1 g of sample to 9-mL aliquots of PBS and preparing up to six tenfold serial dilutions of the suspension. The 10^–6^ dilution (50 μL) was spread onto 90-mm MacConkey (Mac) agar plates (Becton Dickinson, Fair Lawn, NJ) using 10–15 sterilized 3-mm glass beads. Hand swabs in PBS were first mixed to achieve a homogeneous suspension. Half (500 μL) of the suspension was then transferred to sterile microtubes and centrifuged at 2700×*g* for 15 min. After drawing out 450 μL of the supernatant, the remaining sediment suspension was spread onto 90-mm Mac agar plates using sterile beads. Water samples were centrifuged at 2700×*g* for 30 min and 1 mL of the sediment was transferred into a 1.5 mL microcentrifuge tube for further centrifugation at 9800×*g* for 10 min. The pellet was re-suspended in 200 μL of PBS and 50 μL was spread onto 90 mm Mac agar plates. All plates were incubated overnight (18–24 h) at 37 °C in a stationary incubator. The total number of presumptive *E. coli* colonies for each plate was enumerated and used to calculate the number of colony-forming units (CFU) per gram (stool). Hand swabs and water samples were only used to determine presence or absence of resistant bacteria; these were not enumerated. No antibiotics were added to any of the agar plates.

Twelve presumptive *E. coli* isolates [16] were collected from each adult and child sample to total 24 per household. Collection was done using sterile toothpicks. Isolates were transferred into individual wells of 96-well microtiter plates pre-filled with 100 μL of Luria–Bertani broth (Becton Dickinson, Fair Lawn, NJ). The microtiter plates were incubated for 18–24 h. Glycerol [15% (v/v)] was added to each well and plates were frozen at − 20 °C until tested for antibiotic susceptibility.

Prior to testing, a replicate of the original microtiter plate was prepared. A sterile 96-pin replicator was used to transfer ~ 2 µL of thawed culture into sterile microtiter plates containing 100 μL per well of Luria–Bertani broth. The plates were incubated overnight at 37 °C. A break-point assay was conducted by transferring approximately 2 µL of thawed culture onto 150-mm Mac agar plates containing one of nine antibiotics [32 μg/ml ampicillin, Amp; 8 μg/ml ceftazidime, Caz; 32 μg/ml chloramphenicol, Chl; 4 μg/ml ciprofloxacin, Cip; 64 μg/ml kanamycin, Kan; 16 μg/ml streptomycin, Str; 512 μg/ml sulfamethoxazole, Sul; 16 μg/ml tetracycline, Tet; and 16 μg/ml trimethoprim, Tmp (all from Sigma, St. Louis, MO)]. A reference plate (Mac without antibiotic) was prepared for each 96-well plate to confirm cell viability. Isolates were either scored resistant (visible growth) or susceptible (no growth). Partial growth (satellite colonies) relative to the reference plate was considered susceptible. For quality control, 248 presumptive *E. coli* isolates were subjected to biochemical confirmation using Triple Sugar Iron agar (BD Difco™, Sparks, MD) and Motility Indole Ornithine medium (BD Difco™, Sparks, MD). Of these 15 were randomly selected isolates for further confirmation using the API^®^ 20E test kit (bioMérieux^®^ sa, Marcy-l'Etoile, France) following manufacturer’s instructions.

### Data management and analysis

Household survey data were checked for accuracy and completeness within a day of collection and were compiled in a Microsoft Access database (Microsoft, Redmond, WA). Sample data were managed in Microsoft Excel (Microsoft, Redmond, WA). The main outcomes of interest were “load of resistance” (stool samples only), prevalence of resistance and number of resistance patterns. The load of resistance was calculated as a product of the prevalence of resistance (proportion of stool isolates resistant to each antibiotic) and the quantified *E. coli* CFU. This measure was chosen to reflect different reservoir “sizes” of resistant bacteria and to provide greater analytical power to detect modest changes in colonization that may not have been detectable with prevalence values. Load data were log_10_-transformed and analyzed as continuous variables. Linear mixed-effects models fit by the restricted maximum likelihood lme4 package in R [17] were used to identify predictors for the load (log_10_) of resistant *E. coli* in stool samples. Separate models were constructed to predict household-level (adult and child samples) and individual-level (adult or child samples) AMR load. Antibiotic use, water, sanitation, and hygiene-related variables were tested as the main predictors for the carriage of antibiotic resistant *E. coli*. Demographic and environmental variables were additional predictors.

Effects due to household respondents were assumed to be constant across households. Consequently, only a household-specific random intercept (Household ID) was included in household-level models, and an individual-specific random intercept (Respondent ID) in individual-level models. This allowed us to account for variations due to repeated sampling of households and individuals, respectively. Each fixed effect was regressed independently against household or individual AMR load, applying the appropriate random intercept. Variables with *P* < 0·2 in two or more univariable models were included in the multivariable regression analyses. To avert modeling problems resulting from collinearity, a Pearson’s correlation coefficient was used to identify correlated variables (*r* ≥ 0·7) after which one of a pair of correlated variables was removed. Variables with *P* < 0·05 in the multivariable analysis were considered significant predictors of the load of resistant *E. coli.*

## Results

### Survey respondents

**Fig. 1:**
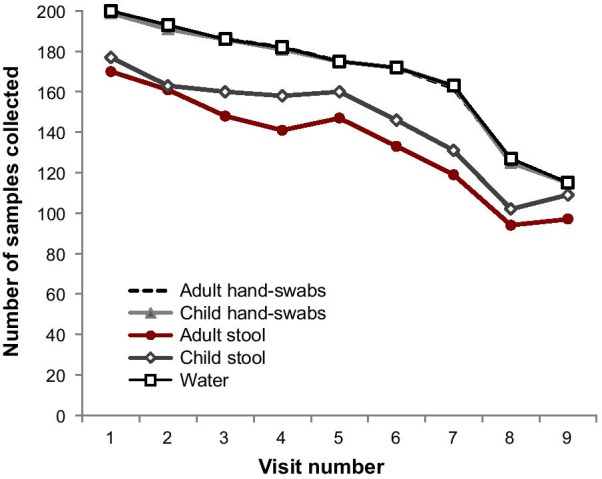
Distribution of 6674 samples (2843 hand swabs, 2341 stool, 1490 water) collected from 200 study households over a 5-month study period. Sample collection on visits 8 and 9 was impacted by temporary migration of study respondents to other areas for holiday festivities. Visits 1 to 8 occurred between August and December 2015 while Visit 9 occurred in January 2016

Eighty-one percent (81%) of the 200 enrolled households participated in seven of the nine visits. Household participation dipped in visits 8 (64%) and 9 (58%) because some study respondents traveled elsewhere for holiday festivities (Fig. 1). In total, 1516 household interview responses were collected and analyzed. Our selection criteria resulted in a predominantly female adult sample (97%) constituting primarily the mothers of the enrolled children (Table 1).

**Table 1:** Baseline characteristics of participating respondents and households (n = 200)

	Counts (%)	Mean (range)	SD
*Respondent characteristics*			
Female	194 (97.0)	–	–
Mother of enrolled child	188 (94.0)	–	–
Age of enrolled child (years)	–	1.6 (0–5)	1.1
Age of enrolled adult (years)	–	28.7 (18–68)	7.8
*Mother's level of education*			
No education	11 (5.5)	–	–
Primary school	119 (59.5)	–	–
High school/vocational training	61 (30.5)	–	–
College/university	9 (4.5)	–	–
*Household population structure*			
Household size	–	5.2 (2–13)	1.9
Members ≥ 18 years	–	2.4 (1–6)	1.1
Members ≤ 5 years	–	1.4 (1–4)	0.6
School-going children	–	1.8 (0–9)	1.6

Frequencies and percentages are provided for categorical values while mean and standard deviation (SD) are provided for continuous values

### Antibiotic use

**Fig. 2:**
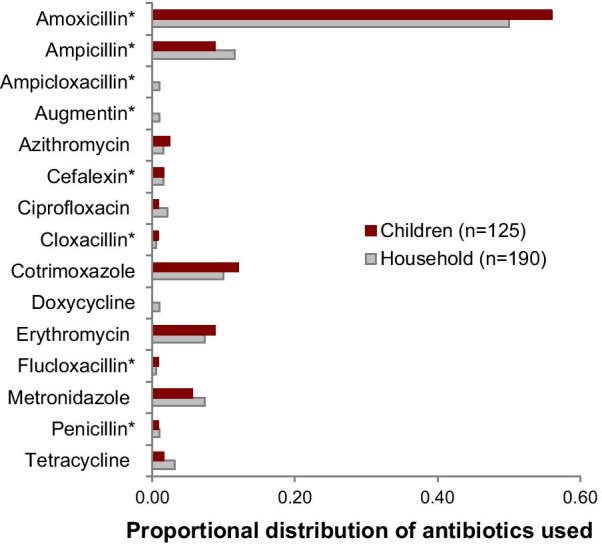
Aggregate distribution of antibiotic use by households and by enrolled children over the study period (includes second antibiotic if use was reported). Asterisk* indicates beta-lactam antibiotic

Ninety-seven (48·5%) of the 200 households reported an antibiotic use episode in at least one visit over the 5-month period. This accounted for 144 episodes, i.e., reported case of antibiotic use by the enrolled adult, enrolled child, and/or other household member, and a total of 190 antibiotic “doses”. Enrolled children had five times the number of episodes reported by enrolled adults (96 vs. 19) and three times that by other household members (96 vs. 29). Episodes by enrolled children represented 67% (96/144) of household antibiotic use episodes. Fifteen different antibiotics were reported over the study period, 53% of which were beta-lactam antibiotics. Amoxicillin was the most used antibiotic within the household (50%; 95/190), followed by ampicillin (12%; 22/190), cotrimoxazole (10%; 19/190), erythromycin and metronidazole (each 7%; 14/190). Enrolled children commonly used amoxicillin (56%; 70/125) and cotrimoxazole (12%; 15/125); Fig. 2. Data collectors confirmed the identities of antibiotics in 74% of reported instances of use.

### Water, sanitation, and hygiene

Most households (92%) accessed water for their daily household needs from protected sources, and 81% spent < 15 min to make a return trip to the main water source (Table 2). Observational data confirmed that of the 166 (83%) households that stored drinking water within the household at baseline, 78.3% used storage containers with narrow openings (< 3 cm), and 89.6% secured their storage containers with lids. Less than half of households (44%) reported treating their water. More households reported water unavailability at enrollment than in subsequent visits (53% vs. 15%). No household owned a toilet; the majority relied on public toilets. The type of toilet used varied by time of day, particularly for flush toilets (48% during the day vs. 7% at night) and buckets/plastic bags (0% during the day vs. 35% at night). Handwashing after toilet use was reportedly high (> 75%) although < 50% of households reported having a designated hand-washing station. On average, 54% of households reported that the enrolled child spent 1 to 12 h outside the household, primarily playing within the household periphery (87%). Of these, 49% also indicated that the child ate soil from the household compound. Household responses on water, sanitation and hygiene questions were consistent between the enrollment visit and subsequent visits except for the question regarding water unavailability i.e. 53% vs. 15% (Table 2).

**Table 2:** Proportion of household responses (mean and 95% confidence interval) for questions regarding water, sanitation, and hygiene-related practices at enrollment (V1) and over nine sampling visits

Water			Sanitation			Hygiene		
	V1	Mean [95% CI]		V1	Mean [95% CI]		V1	Mean [95% CI]
*Type of water source*^a^			*Type of toilet used during daytime*			*Washed hands after urination only [last 3 toilet visits]*		
Private-protected	0.40	0.36 [0.29, 0.43]	Flush toilet	0.45	0.48 [0.46, 0.50]	1 out of 3 events	0.16	0.09 [0.06, 0.12]
Public-protected	0.52	0.61 [0.53, 0.69]	Ventilated-improved pit	0.03	0.04 [0.03, 0.05]	2 out of 3 events	0.20	0.14 [0.10, 0.18]
Private-unprotected	0.01	0.01 [0.00, 0.02]	Pit with slab	0.41	0.34 [0.32, 0.36]	All 3 events	0.64	0.77 [0.70, 0.84]
Public-unprotected	0.09	0.03 [− 0.01, 0.07]	Traditional pit latrine^d^	0.12	0.13 [0.12, 0.14]	^c^Washed with soap^c^ [yes]	0.80	0.83 [0.79, 0.87]
*Time taken to go, collect water, and return*			*Households sharing same daytime toilet facility*			*Washed hands after defecation [last 3 toilet visits]*		
5 min or less	0.28	0.19 [0.16, 0.22]	1–10	0.21	0.20 [0.19, 0.21]	1 out of 3 events	0.08	0.02 [0.00, 0.04]
5–9 min	0.34	0.44 [0.39, 0.49]	11–50	0.30	0.26 [0.24, 0.28]	2 out of 3 events	0.18	0.10 [0.06, 0.14]
10–14 min	0.18	0.18 [0.15, 0.21]	51–100	0.44	0.33 [0.23, 0.43]	All 3 events	0.74	0.88 [0.83, 0.93]
≥ 15 min	0.20	0.19 [0.16, 0.22]	> 100	0.06	0.21 [0.11, 0.31]	Washed with soap^c^ [yes]	0.74	0.90 [0.85, 0.95]
*Water unavailable at source in the last 14 days*			*Frequency of toilet cleaning during the last 14 days*			*Washed hands before feeding baby [last 3 feeding events]*		
1–6 days	0.44	0.13 [0.04, 0.22]	Daily	0.47	0.56 [0.53, 0.59]	1 out of 3 events	0.09	0.05 [0.04, 0.06]
7–14 days	0.08	0.02 [0.01, 0.03]	Alternate days	0.21	0.16 [0.13, 0.19]	2 out of 3 events	0.23	0.12 [0.07, 0.17]
*Methods used to make water safer to drink*^b^			Occasionally	0.26	0.23 [0.22, 0.24]	All 3 events	0.67	0.83 [0.77, 0.89]
Boiling	0.65	0.52 [0.47, 0.57]	*Location of daytime toilet facility*			Washed with soap^c^ [yes]	0.69	0.86 [0.81, 0.91]
Chemical cleansers	0.54	0.62 [0.57, 0.67]	Within HH compound	0.30	0.32 [0.31, 0.33]	*Location of hand-washing station*		
*Frequency of water treatment in last 14 days*^b^			Outside HH compound	0.70	0.68 [0.67, 0.69]	Within household compound	0.25	0.25 [0.19, 0.31]
Every collection	0.71	0.75 [0.70, 0.80]	*Type of toilet used during the night*			Around kitchen area	0.32	0.45 [0.41, 0.49]
Occasionally	0.29	0.25 [0.20, 0.30]	Flush toilet	0.16	0.07 [0.04, 0.1]	Elsewhere [excl. kitchen/toilet]	0.46	0.37 [0.30, 0.44]
*Last household water treatment*^b^			Ventilated-improved pit	0.03	0.04 [0.03, 0.05]	Nonspecific	0.61	0.59 [0.56, 0.62]
Today/yesterday	0.21	0.26 [0.23, 0.29]	Pit with concrete slab	0.39	0.37 [0.36, 0.38]	*Time spent by the enrolled child outside household*		
2–6 days ago	0.67	0.63 [0.58, 0.68]	Traditional pit latrine	0.12	0.13 [0.12, 0.14]	1–2 h	0.15	0.14 [0.13, 0.15]
≥ 7 days ago	0.11	0.11 [0.08, 0.14]	Bucket/plastic bags	0.29	0.35 [0.32, 0.38]	3–4 h	0.21	0.19 [0.16, 0.22]
			Open areas around HH	0.02	0.03 [0.02, 0.04]	5 + h	0.30	0.21 [0.16, 0.26]
						*Places where the enrolled child spends time*		
						Plays in school compound	0.00	0.01 [− 0.03, 0.05]
						Within household environment	0.93	0.87 [0.83, 0.91]
						Relative in another household	0.07	0.12 [0.09, 0.15]
						Enrolled child eats soil [yes]	0.55	0.49 [0.37, 0.61]

Two hundred households were enrolled

*HH *household

^a^A protected source prevents water contamination by the environment e.g., a source covered with a concrete slab or a completely covered tank

^b^Applies only to households that reported treating water (mean = 44%)

^c^Applies to those who reported washing hands mean = 80% (urination), 98% (defecation) or 87% (feeding baby)

^d^Single pit covered by a wooden, earthen, or concrete slab with a drop hole

### Antimicrobial resistance

**Fig. 3:**
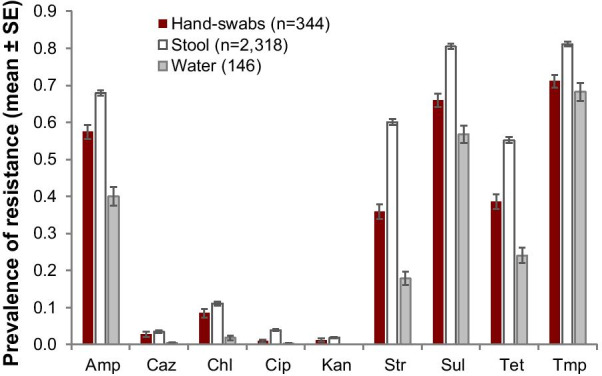
Prevalence of resistant *E. coli* (mean and standard error) in stool, hand swabs, and water samples. Stool and hand swab values are pooled estimates for adults and children. Amp, ampicillin; Caz, ceftazidime, Chl, chloramphenicol; Cip, ciprofloxacin; Kan, kanamycin, Str, streptomycin; Sul, sulfamethoxazole; Tet, tetracycline; Tmp, trimethoprim

Presumptive *E. coli* were isolated from 99·0% of stool (n = 2341), 12·1% hand (n = 2843) and 9·8% of water (n = 1490) samples. Phenotypic tests for 248 isolates confirmed that our selection criteria for *E. coli* was reliable (99·2% accuracy). Isolates (stool n = 27,451, hand swabs n = 3639, water n = 2952) were tested against nine antibiotics from seven antibiotic classes. Of these, 23,981 (87·4%) stool isolates, 3020 (83·0%) hand swab isolates and 2354 (79.7%) water isolates were resistant to at least one antibiotic; 80% percent of all presumptive *E. coli* isolates were resistant to ≥ 3 antibiotic classes. The prevalence of resistance to ampicillin, streptomycin, sulfamethoxazole, tetracycline, and trimethoprim was > 15% across all sample types and > 50% among stool samples. Resistance to ceftazidime, chloramphenicol, ciprofloxacin, and kanamycin was < 15% across all specimen types (Fig. 3).

**Fig. 4:**
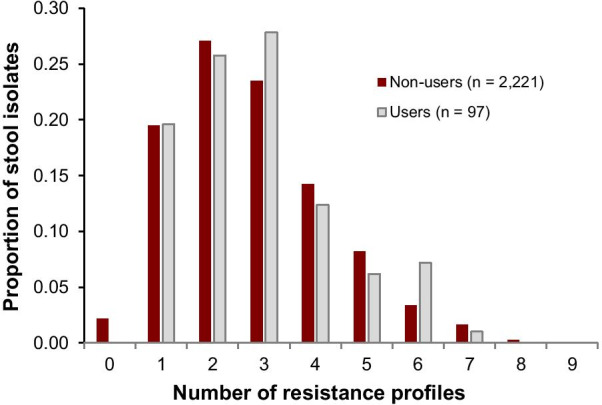
Aggregate distribution of AMR profiles identified in 2318 stool samples. This includes individuals who reported using (users) or not using an antibiotic (non-users) during the study period; not all (non)users provided stool samples

A total of 148 unique resistance profiles were identified from the isolates collected in this study. A “penta-resistant” profile AmpStrSulTetTmp predominated in stool isolates (31·3%) and hand swabs (19·6%), while SulTmp was the most abundant in isolates from water (20·4%; Additional file 3). Half of all stool samples had two or three resistance profiles of varying antibiotic combinations. The distribution of unique resistance phenotypes in stool samples was similar for individuals that reported using antibiotics (users) and those that did not (non-users); Fig. 4. Pan-resistant isolates (resistant to the nine antibiotics tested) were identified in one adult and one child sample from two different households. These individuals reported not having used antibiotics in the two weeks preceding their sample collection.

**Fig. 5:**
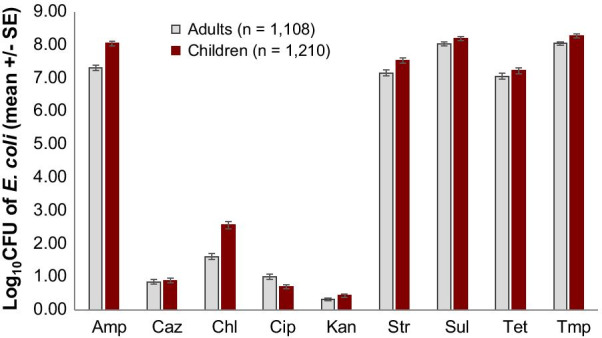
Load of resistant *E. coli* (mean and standard error) in adult and child stool samples. Amp, ampicillin; Caz, ceftazidime, Chl, chloramphenicol; Cip, ciprofloxacin; Kan, kanamycin, Str, streptomycin; Sul, sulfamethoxazole; Tet, tetracycline; Tmp, trimethoprim

The average load of resistant *E. coli* in stool ranged between 1·4 × 10^7^ and 1·5 × 10^8^ CFU/g for the highly prevalent resistance phenotypes (Amp, Str, Sul, Tet and Tmp), and between 0·7 × 10^1^ and 1·3 × 10^2^ CFU/g for low-prevalence phenotypes (Caz, Chl, Cip and Kan). The distribution of resistant *E. coli* in adult and child stool samples was similar although on average, children had marginally higher loads of ampicillin and chloramphenicol-resistant *E. coli* (Fig. 5).

### Predictors of antimicrobial resistance

Univariable analyses identified water, sanitation, and antibiotic use variables as potential predictors (*P* < 0·2) of both prevalence and load of resistant *E. coli* at household and individual levels. When these predictors were added to multivariable regression models, the level of significance (*P* < 0·05) varied depending on the antibiotic tested, and the level of analysis i.e. household or individual (Tables 3, 4, 5). Antibiotic use within the household was not a significant risk factor for resistance to any of the nine antibiotics tested in adults and was only associated with resistance to two antibiotics in children (Sul, β = 0·46 log-increase in load; Tmp, β = 0·42; Table 5).

**Table 3:** Multivariable regression analysis for antimicrobial resistance load (Log_10_ CFU) at the household level

*Predictor*	Ampicillin	Streptomycin	Sulfamethoxazole	Tetracycline	Trimethoprim
	β [95% CI]	β [95% CI]	β [95% CI]	β [95% CI]	β [95% CI]
Night toilet type					
Ventilator improved pit	0.46 [− 0.34, 1.26]	0.32 [− 0.59, 1.23]	0.07 [− 0.59, 0.74]	− 0.27 [− 1.22, 0.67]	− 0.06 [− 0.69, 0.57]
Pit with concrete slab	0.49 [0.00, 0.99]	0.50 [− 0.06, 1.07]	0.13 [− 0.28, 0.54]	− 0.13 [− 0.71, 0.46]	− 0.04 [− 0.43, 0.35]
Traditional pit latrine^a^	0.03 [− 0.58, 0.63]	0.25 [− 0.44, 0.94]	− 0.07 [− 0.57, 0.43]	*− 0.79 [− 1.51, − 0.08]*	− 0.07 [− 0.54, 0.40]
Bucket/plastic	0.26 [− 0.23, 0.75]	0.23 [− 0.32, 0.79]	− 0.06 [− 0.47, 0.34]	*− 0.62 [− 1.19, − 0.04]*	− 0.24 [− 0.62, 0.15]
No facilities/open field	0.46 [− 0.29, 1.22]	0.03 [− 0.82, 0.88]	− 0.02 [− 0.64, 0.60]	− 0.71 [− 1.59, 0.18]	*− 0.64 [− 1.23, − 0.04]*
Handwashing with soap	*0.20 [0.01, 0.40]*	0.21 [− 0.01, 0.43]	0.07 [− 0.09, 0.23]	0.19 [− 0.03, 0.42]	0.10 [− 0.06, 0.25]
Handwashing facility location					
Toilet within premises	0.14 [− 0.15, 0.43]	0.13 [− 0.19, 0.46]	0.10 [− 0.13, 0.34]	0.25 [− 0.09, 0.59]	0.17 [− 0.05, 0.4]
Elsewhere on premises^b^	*0.47 [0.20, 0.73]*	0.28 [− 0.02, 0.58]	*0.35 [0.13, 0.57]*	*0.36 [0.05, 0.67]*	*0.22 [0.01, 0.43]*
No designated place	*− 0.28 [− 0.55, − 0.02]*	− 0.13 [− 0.44, 0.17]	0.04 [− 0.18, 0.26]	0.26 [− 0.05, 0.58]	− 0.21 [− 0.42, 0.00]
Enrolled child					
Time spent outside (h)	0.00 [− 0.05, 0.05]	− 0.01 [− 0.08, 0.05]	0.01 [− 0.03, 0.06]	0.03 [− 0.03, 0.09]	0.00 [− 0.04, 0.04]
Eats soil	*0.51 [0.26, 0.77]*	*0.39 [0.10, 0.68]*	*0.27 [0.06, 0.48]*	*0.54 [0.24, 0.84]*	*0.28 [0.08, 0.48]*
Rainfall (per mm)	*− 2.56 [− 3.60, − 1.52]*	*− 1.23 [− 2.40, − 0.06]*	− 0.57 [− 1.42, 0.28]	1.18 [− 0.03, 2.40]	*− 1.19 [− 2.00, − 0.37]*
Mother’s education level					
Primary school	− 0.52 [− 1.17, 0.14]	− 0.72 [− 1.48, 0.05]	− 0.25 [− 0.82, 0.32]	− 0.11 [− 0.91, 0.68]	0.02 [− 0.50, 0.53]
High school	*− 0.87 [− 1.58, − 0.16]*	*− 0.91 [− 1.73, − 0.08]*	− 0.29 [− 0.90, 0.32]	− 0.26 [− 1.11, 0.60]	0.02 [− 0.53, 0.57]
College	− 0.93 [− 1.90, 0.05]	*− 1.40 [− 2.53, − 0.26]*	− 0.32 [− 1.16, 0.52]	− 0.70 [− 1.88, 0.47]	0.19 [− 0.57, 0.95]
Respondent age (years)	*− 0.02 [− 0.03, − 0.02]*	*− 0.01 [− 0.02, 0.00]*	0.00 [− 0.01, 0.00]	0.00 [− 0.01, 0.00]	*− 0.01 [− 0.01, 0.00]*

Only variables with *P* < 0·2 in the univariable mixed-effects model were included in the multivariable model. Regression estimates for increasing or decreasing antimicrobial load (positive or negative β coefficients, respectively) and 95% confidence intervals with *P* < 0·05 are shown in bold. Only variables with significant values are shown. See Additional file 4 for complete table

^a^Single pit covered by a wooden, earthen or concrete slab with a drop hole

^b^Other than at a toilet facility or the household kitchen

**Table 4:** Multivariable regression analysis for antimicrobial resistance load (Log_10_ CFU) at the adult level (≥ 18 years)

Predictor	Ampicillin	Streptomycin	Sulfamethoxazole	Tetracycline	Trimethoprim
	β [95% CI]	β [95% CI]	β [95% CI]	β [95% CI]	β [95% CI]
Main water source^a^					
Public-protected	− 0.47 [− 0.89, − 0.05]	− 0.30 [− 0.75, 0.16]	− 0.08 [− 0.39, 0.22]	− 0.19 [− 0.65, 0.27]	-0.26 [-0.55, 0.03]
Private-unprotected	0.36 [− 1.88, 2.60]	0.49 [− 1.88, 2.86]	0.12 [− 1.52, 1.76]	0.83 [− 1.57, 3.23]	0.16 [-1.46, 1.78]
Public-unprotected	− 0.27 [− 1.25, 0.70]	− 0.09 [− 1.12, 0.95]	− 0.29 [− 1.00, 0.42]	0.24 [− 0.80, 1.29]	-0.34 [-1.04, 0.35]
Handwashing after urination	0.07 [− 0.08, 0.23]	− 0.03 [− 0.19, 0.14]	0.04 [− 0.08, 0.15]	0.18 [0.01, 0.34]	0.04 [-0.07, 0.15]
Handwashing facility location					
Toilet within premises	0.23 [− 0.18, 0.64]	0.12 [− 0.31, 0.56]	0.05 [− 0.24, 0.35]	0.23 [− 0.21, 0.67]	0.11 [-0.17, 0.40]
Elsewhere on premises	*0.51 [0.15, 0.88]*	0.20 [− 0.19, 0.59]	*0.30 [0.04, 0.57]*	*0.42 [0.02, 0.81]*	*0.29 [0.03, 0.54]*
No designated place	− 0.48 [− 0.85, − 0.11]	− 0.24 [− 0.64, 0.16]	− 0.07 [− 0.34, 0.20]	0.23 [− 0.17, 0.63]	-0.19 [-0.45, 0.06]
Enrolled child eats soil	*0.41 [0.02, 0.79]*	0.24 [− 0.17, 0.65]	*0.30 [0.02, 0.58]*	0.32 [− 0.09, 0.74]	0.23 [-0.03, 0.50]
Rainfall (per mm)	*− 3.26 [− 4.88, − 1.65]*	− 1.11 [− 2.82, 0.59]	− 0.65 [− 1.84, 0.54]	0.47 [− 1.26, 2.20]	*-1.79 [-2.97, -0.61]*
School children (counts)	0.03 [− 0.11, 0.18]	− 0.02 [− 0.18, 0.14]	− 0.13 [− 0.23, − 0.03]	− 0.07 [− 0.23, 0.09]	-0.10 [-0.20, -0.01]
Mother’s education level					
Primary school	− 0.74 [− 1.69, 0.21]	− 0.58 [− 1.62, 0.46]	0.07 [− 0.59, 0.73]	− 0.21 [− 1.26, 0.82]	0.26 [-0.33, 0.86]
High school	− 1.17 [− 2.15, − 0.19]	− 0.76 [− 1.84, 0.32]	− 0.02 [− 0.71, 0.66]	− 0.44 [− 1.51, 0.64]	0.28 [-0.33, 0.89]
College	− 1.78 [− 3.09, − 0.47]	− 1.42 [− 2.85, 0.02]	− 0.52 [− 1.44, 0.39]	− 1.53 [− 2.97, − 0.10]	0.02 [-0.80, 0.84]
Adult age (years)	0.00 [− 0.03, 0.03]	0.02 [− 0.01, 0.05]	*0.03 [0.01, 0.05]*	0.02 [− 0.01, 0.05]	*0.03 [0.01, 0.05]*

Only variables with *P* < 0·2 in the univariable mixed-effects model were included in the multivariable model. Regression estimates for increasing or decreasing antimicrobial load (positive or negative β coefficients, respectively) and 95% confidence intervals with *P* < 0·05 are shown in bold. Only variables with significant values are shown. See Additional file 5 for complete table

^a^A protected source prevents contamination of water by the environment e.g. a source covered with a concrete slab or a completely covered tank

^b^Other than at a toilet facility or the household kitchen

**Table 5:** Multivariable regression analysis for antimicrobial resistance load (Log_10_ CFU) for children aged 0–5 years

Predictors	Ampicillin	Streptomycin	Sulfamethoxazole	Tetracycline	Trimethoprim
	β [95% CI]	β [95% CI]	β [95% CI]	β [95% CI]	β [95% CI]
Night toilet type					
Ventilator improved pit	0.03 [− 0.95, 1.00]	0.46 [− 0.73, 1.64]	0.25 [− 0.65, 1.16]	− 0.59 [− 1.81, 0.64]	0.02 [− 0.81, 0.86]
Pit with concrete slab	0.24 [− 0.38, 0.86]	0.41 [− 0.35, 1.17]	0.18 [− 0.39, 0.76]	− 0.38 [− 1.17, 0.41]	0.06 [− 0.48, 0.59]
Traditional pit latrine^a^	0.00 [− 0.75, 0.75]	0.25 [− 0.66, 1.16]	0.14 [− 0.56, 0.84]	*− 1.39 [− 2.33, − 0.45]*	0.20 [-0.44, 0.85]
Bucket/plastic	− 0.08 [− 0.69, 0.53]	0.19 [− 0.55, 0.93]	− 0.01 [− 0.57, 0.56]	*− 0.99 [− 1.77, − 0.21]*	-0.17 [-0.70, 0.35]
No facilities/open field	− 0.07 [− 1.05, 0.91]	− 0.43 [− 1.63, 0.76]	− 0.19 [− 1.09, 0.72]	− 1.28 [− 2.56, 0.00]	-0.78 [*−* 1.63, 0.06]
Handwashing after urination	*0.20 [0.03, 0.38]*	0.06 [− 0.16, 0.27]	0.12 [− 0.04, 0.28]	0.08 [− 0.14, 0.31]	*0.19 [0.04, 0.34]*
Handwashing facility location					
Toilet within premises	0.12 [− 0.23, 0.47]	0.19 [− 0.24, 0.62]	0.17 [− 0.16, 0.50]	0.28 [− 0.18, 0.74]	0.28 [*−* 0.03, 0.58]
Elsewhere on premises^b^	*0.45 [0.12, 0.78]*	0.39 [− 0.01, 0.79]	*0.35 [0.05, 0.66]*	0.26 [− 0.16, 0.68]	0.28 [0.00, 0.57]
Child eats soil	*0.59 [0.27, 0.91]*	0.52 [0.14, 0.91]	0.22 [− 0.08, 0.52]	*0.80 [0.40, 1.21]*	0.25 [*− *0.03, 0.52]
Rainfall (per mm)	*− 2.02 [− 3.28, − 0.76]*	− 1.26 [− 2.80, 0.27]	− 0.69 [− 1.85, 0.47]	1.74 [0.07, 3.40]	*− *0.82 [*− *1.91, 0.27]
Altitude (10 m increments)	*− 0.01 [− 0.01, 0.00]*	0.00 [− 0.01, 0.01]	*− 0.01 [− 0.01, 0.00]*	− 0.01 [− 0.01, 0.00]	*− 0.01 [− 0.01, 0.00]*
Household used antibiotic	0.39 [− 0.07, 0.84]	0.22 [− 0.34, 0.77]	*0.46 [0.04, 0.88]*	0.10 [− 0.49, 0.69]	*0.42 [0.02, 0.81]*

Only variables with *P* < 0·2 in the univariable mixed-effects model were included in the multivariable model. Regression estimates for increasing or decreasing antimicrobial load (positive or negative β coefficients, respectively) and 95% confidence intervals with *P* < 0·05 are shown in bold. Only variables with significant values are shown. See Additional file 6 for complete table

^a^Single pit covered by a wooden or earthen slab with a drop hole

^b^Other than at a toilet facility or the household kitchen

Enrolled children eating soil (β = 0·27 to 0·80 log increase) and presence of a common hand-washing station within the housing block (β = 0·22 to 0·51) were both associated with increased load of antimicrobial-resistant *E. coli* at both household and individual levels. The presence of a common hand-washing station was associated with increased load at the level of household (Tmp, Sul, Tet, Amp, β = 0·22 to 0·47), among adults (Tmp, Sul, Tet, Amp, β = 0·29 to 0·51), and children (Sul, Amp, β = 0·35 to 0·45).

Rainfall was the single best predictor for decreased load (1·19 to 3·26 log) of antibiotic-resistant *E. coli* at the level household (Tmp, Str, Amp, β = − 1·19 to − 2·56; Table 2), among adults (Tmp, Amp, β = − 1·79 to − 3·26; Table 3) and children (Amp, β = − 2·02) levels (Table 5). Household elevation (10 m increments) was associated with slight load reductions among children (Amp, Sul, Tri, β = − 0·10).

## Discussion

During a 2016 survey of antibiotic use in Kibera, 87% of respondents reported using an antibiotic in the preceding 12 months [11]. In this study, half of the enrolled households reported using an antibiotic within a 5-month period, consistent with the high burden of disease reported in this community [3, 10, 18]. A World Health Organization survey of 12 low- and middle-income countries found that 35–76% of respondents had used antibiotics in the previous six months [19] in contrast to lower levels of use reported in wealthier countries [20]. Nevertheless, we found no consistent association between the reported use of antibiotics in our study and the abundance, prevalence, or diversity of antibiotic-resistant *E. coli* in this population despite the common use of oral formulations (Fig. 2) that should selectively favor antibiotic-resistant intestinal *E. coli*.

The lack of clear association between antibiotic use and prevailing levels of AMR does not imply that there is no causal relationship, rather that in this community, carriage of resistant bacteria changes little in response to incremental changes in antibiotic use. Instead, our analysis identified environmental and sanitation variables as predictive for the abundance of antibiotic-resistant *E. coli* in Kibera households (Table 3) and among individual household members (Tables 4, 5). Poor sanitation and environmental contamination likely play a dual role of increasing disease burden and demand for antibiotics (keeping selective pressure high at the community level), while also disseminating antibiotic-resistant bacteria within and between households. For example, approximately 10% of all hand swabs and water samples confirmed contamination with resistant bacteria (Fig. 3).

Sanitation-related factors have been implicated in the spread of infectious diseases [21–24], child malnutrition and/or stunting [25], cognitive deficiencies in children, and poor school attendance [21, 26]. Their role in the spread of antibiotic-resistant bacteria has also been postulated [27, 28]. Households in our study had no toilets within the premises, but generally had access to some form of public toilet facility, particularly during daytime [15]. Nevertheless, these public toilets were shared by at least 10 other households. Personal security concerns markedly reduces use of sanitation facilities at night [29], forcing households to improvise by using buckets, plastic bags (“flying toilets”) or open spaces outside the household to dispose of feces. These alternative disposal options have been documented by others [14, 26] and contribute to significant environmental contamination [30].

Fecal environmental contamination likely explains why the variable “children eating soil” was a significant predictor for increased individual and household AMR load. This environmental connection is consistent with the negative association between the load of antibiotic-resistant *E. coli* in stool samples and rainfall, as runoff can dilute or remove fecal sources of bacteria in the environment (also consistent with the observed elevation correlation for AMR prevalence in isolates from children), or discourage outdoor activities and thus reduce contact with contaminated environments. Furthermore, wet seasons are generally associated with lower ambient temperatures, which may reduce the environmental load of bacteria.

Unfortunately, when hand-washing stations were used by multiple households within the housing block they were a risk factor for a higher load of antibiotic-resistant *E. coli*. While this correlation seems counter-intuitive, it is consistent with these stations serving as fomites. Moist surfaces around wash stations favor bacterial survival and proliferation, and the inconsistent availability of soap likely contributes to this outcome. This interpretation is supported by the association between lack of a hand-washing station and lower Amp resistance, and by behavioral practices for which handwashing after use of public toilets was associated with increased Amp-resistant *E. coli* in children and increased Tet-resistant *E. coli* in adults.

Like many communities in low-income countries [31–33], residents of Kibera have easy access to a limited diversity of antibiotics. For example, oral formulations of beta-lactams were the most used, with amoxicillin accounting for 50 and 56% of antibiotics used by households and children, respectively. Amoxicillin is a broad-spectrum antibiotic that is used to treat acute respiratory and febrile illnesses, which are prevalent in this community [3] and for which residents report using antibiotics [11]. As might be expected, children consumed the most antibiotics within the household perhaps supporting the only positive correlation detected between antibiotic use and AMR (i.e., sulfamethoxazole and trimethoprim resistance) among children (Table 4). Sulfa drugs are used to treat malaria and this practice has been correlated with the load of sulfamethoxazole-resistant *E. coli* in children [34]. While Kibera is not situated in a malaria-endemic area, travel to malaria-endemic areas is common among residents [35], and might contribute to this practice.

As a community, Kibera suffers from poor sanitation and a dense population, conditions that favor transmission of infectious diseases. It is unclear what proportion of antibiotic use in this setting is justified. Nevertheless, antibiotics likely provide a much-needed health benefit while inadvertently selecting for antibiotic-resistant bacteria. AMR transmission is a density-dependent process. Thus, when resistant bacteria in environments with poor sanitation are enriched from antibiotic use, ideal conditions for a steady production of antibiotic-resistant bacteria are achieved. 

We acknowledge several limitations of our study. Firstly, self-reported data, inaccurate recall and biased responses could have increased variance in our results. Additionally, by sampling individuals who were available at home, our results may not be generalized to adult males and school-going children. Given that most enrolled adults were female household heads with extensive knowledge of household practices, and that household interactions facilitate “sharing” of germs, we surmise that our data were a reasonable representation of enrolled households. Secondly, we relied on colony morphology as the primary method to select presumptive *E. coli* isolates for analysis. Colony morphology, while not a reliable diagnostic for species identity, was 99.2% consistent with *E. coli* based on a random subset of 248 isolates. We have successfully used this method for selecting *E. coli* for high-throughput assessment of antibiotic resistance in other studies [16, 36–38]. Thirdly, the "breakpoint" assay is not considered a diagnostic tool in a clinical microbiology lab but provides a low-cost means to analyze many isolates. We have assessed the validity of this method genotypically [36] and phenotypically [38].

## Conclusion

Kibera experiences sanitation challenges that promote disease transmission and demand for antibiotics within a highly dense population. Under these conditions it is likely that selection of antibiotic-resistant bacteria within the gut, their disposal and enrichment within the environment, and recurrent transmission to humans occurs continuously. For communities that suffer such scenarios, the fight against antibiotic-resistant bacteria will require significant reduction in the burden of infectious disease coupled with markedly improved sanitation at the household and community levels.

## Supplementary information

**Additional file 1.** Water, Sanitation and Hygiene Questionnaire.

**Additional file 2.** Antibiotic use Questionnaire.

**Additional file 3.** Nine of the most abundant (found in > of all E. coli isolates) resistance phenotypes out of 148 unique combinations. The number of isolates and their proportional representation (%) of the total are shown. Profile marked with an asterisk (*) denotes the penta-resistant phenotype.

**Additional file 4.** Multivariable regression analysis for antimicrobial resistance load (Log_10_ CFU) at the household level. Only variables with *P* < 0·2 in the univariable mixed-effects model were included in the multivariable model. Regression estimates (β) and 95% confidence intervals with *P* < 0·05 are shown in bold. *P* = 0·00 indicates *P* < 0·01.

**Additional file 5.** Multivariable regression analysis for antimicrobial resistance load (Log_10_ CFU) at the adult level (≥18 years). Only variables with *P* < 0·2 in the univariable mixed-effects model were included in the multivariable model. Regression estimates (β) and 95% confidence intervals with *P* < 0·05 are shown in bold. *P* = 0·00 indicates *P* < 0·01.

**Additional file 6.** Multivariable regression analysis for antimicrobial resistance load (Log_10_ CFU) for children aged 0–5 years. Only variables with *P* < 0·2 in the univariable mixed-effects model were included in the multivariable model. Regression estimates (β) and 95% confidence intervals with *P* < 0·05 are shown in bold. *P* = 0·00 indicates *P* < 0·01.

## Data Availability

The datasets used and/or analyzed during the current study are available from the corresponding author on reasonable request.

## Abbreviations

AMR: Antimicrobial resistance
CDC: Centers for Disease Control and Prevention
CFU: Colony-forming units
KEMRI: Kenya Medical Research Institute
PBIDS: Population-based infectious disease surveillance
PBS: Phosphate-buffered saline
