# Supplementary material for: Carriage of antimicrobial-resistant bacteria in a high-density informal settlement in Kenya is associated with environmental risk-factors
Source: Antimicrob Resist Infect Control. 2021 Jan 22;10:18. doi: 10.1186/s13756-021-00886-y (PMC7821723; doi:10.1186/s13756-021-00886-y)
Supplement: Supplementary file 1 — Additional file 1. Water, Sanitation and Hygiene Questionnaire. [file 13756_2021_886_MOESM1_ESM.docx]

**Additional file 1: Water Sanitation and Hygiene Questionnaire**

1. During the last 2 weeks, what was the MAIN source of drinking water for members of this household?

Tap water

Well

Rainwater collection

Bottled water or sachet water

Cart with small tank/drum

Other, specify.

- 1. Was this source of drinking water private, public, protected or unprotected?

Private/Protected

Public/ Protected

Private/UnProtected

Public/Unprotected

- 1. How long did it take to go to this source of drinking water, get water, and come back? (minutes)

- 1. In the last 2 weeks, has water from this source been available every single day? Yes/No
  2. If no, how many days has water been unavailable from this source? (days)
  3. Would you rate the safety of drinking water from this source as very safe, safe or unsafe?

- 1. Did you do anything to make your water safer to drink? Yes/No
     1. What did you do to the water to make it safer to drink?

Boil

Add chemicals

Use a water filter

Solar disinfection

Let it stand and settle

Did not treat the water

Other, specify

- - - 1. How often did you treat your drinking water using this method? ***(CHECK ONE***)

Every time it was collected

Once in a while/occasionally

- - - 1. When was the last time you treated your drinking water using this method?

Today/yesterday

More than two days ago but less than one week ago

More than one week ago

1. During the last two weeks, was there a place or a facility that members of this household could constantly access for their toilet needs DURING THE DAY? Yes/No
   1. What kind of facility did members of this household commonly have/what did members of this household do to meet their toilet needs during the day?

Flush or pour/flush toilet or latrine

Improved pit latrine

Traditional pit latrine

Composting/dry toilet or latrine

Hanging toilet/hanging latrine

Bucket latrine (where feces are manually removed)

There are no facilities, field

There are no facilities, plastic bags

Other, specify

- 1. If it is a place/facility, where is this place or facility located? ***(CHECK ONE)***

Inside or attached to dwelling

Elsewhere on premises

Outside premises

- 1. Did this household share this place/facility with other households during the last two weeks? Yes/No
     1. Approximately how many households would you say shared this place or facility during the last two weeks?
  2. Was this place/facility cleaned over the last two weeks? Yes/No
     1. Approximately how many times do you think this place or facility was cleaned over the last two weeks?
  3. Was this place/facility also available AT NIGHT? Yes/No
     1. What kind of facility did members of this household commonly have/what did they do to meet their toilet needs during the night?

Flush or pour/flush toilet or latrine

Improved pit latrine

Traditional pit latrine

Composting/dry toilet or latrine

Hanging toilet/hanging latrine

Bucket latrine (where feces are manually removed)

There are no facilities, field

There are no facilities, plastic bags

Other, specify

1. Please tell me:
   1. The last three times you went for a short call, were you able to wash your hands on all three occasions? Yes/No
      1. In how many of the three occasions were you able to wash your hands?

None

One out of three

Two out of three

- - 1. Did you use soap every time you washed your hands? Yes/No
  1. The last three times you went for a long call, were you able to wash your hands on all three occasions? Yes/No
     1. In how many of the three occasions were you able to wash your hands?

None

One out of three

Two out of three

- - 1. Did you use soap every time you washed your hands? Yes/No
  1. The last three occasions before feeding the child, were you able to wash your hands on all three occasions? Yes/No
     1. In how many of the three occasions were you able to wash your hands?

None

One out of three

Two out of three

- - 1. Did you use soap every time you washed your hands? Yes/No
  1. Have you washed your hands for any reason today or yesterday? Yes/No

1. Please mention all the places that members of this household could wash their hands in the last two weeks ***(CHECK ALL THAT APPLY)***

In or near toilet facility inside household

In or near toilet facility outside household but on premises

In or near toilet facility outside premises

In or near kitchen

Elsewhere on premises

Outside premises

No specific place/anywhere

Other, specify

1. During the last two weeks, did this (enrolled) child regularly spend most of the day playing outside this household or being under the supervision of someone else? Yes/No
   1. Approximately how many hours in a day was the child away from this household playing or being supervised by someone else?
   2. Where did the child commonly go to play or be supervised by someone else?

School compound

Within the household environment

Relative living in a different household

Other, specify

1. Did (enrolled child’s name) eat soil or earth from any source (for example, walls of mud houses, the market or the yard) during the last two weeks? Yes/No ***(END SURVEY)***
