# Supplementary material for: Carriage of antimicrobial-resistant bacteria in a high-density informal settlement in Kenya is associated with environmental risk-factors
Source: Antimicrob Resist Infect Control. 2021 Jan 22;10:18. doi: 10.1186/s13756-021-00886-y (PMC7821723; doi:10.1186/s13756-021-00886-y)
Supplement: Supplementary file 2 — Additional file 2. Antibiotic use Questionnaire. [file 13756_2021_886_MOESM2_ESM.docx]

**Additional file 2: Antibiotic use Questionnaire**

1. Since our last visit two weeks ago, has ANYONE in this household taken medicine to treat any illness? Yes/No
   1. When was the last time that ANY household member(s) took medicine to treat any illness in the last 14 days?

Today

2-3 days ago

>4 days but within the last 7 days

> 7 days ago

- 1. Is it YOU (enrolled ADULT), the enrolled CHILD (Name child) or (AN)OTHER HOUSEHOLD MEMBER(S) that has taken medicine since our last visit? ***(CHECK ALL THAT APPLY)***

Respondent (enrolled adult)

Study child

Other household members

- 1. Is ANYONE in this household on HIV or TB medication? **(CHECK ONE)** Yes/No
     1. How many members would you say are on HIV or TB medication?

1. Please tell me (for each person listed above):
   1. For which illness(es) did you/they need to use medication? ***(CHECK ALL THAT APPLY)***

Runny nose/nasal congestion

Cough

Headache

Fever

Vomiting

Diarrhea

Skin wounds

Malaria

HIV

Tuberculosis

Other, specify

***For each ILLNESS mentioned ASK:***

- 1. What medicine(s) did you/they use for this illness? (**A*sk to be shown medication if still available.*** ***Probe “what else” until nothing further is mentioned and check all that apply; end survey if no antibiotic is shown/mentioned***)

Antibiotics ***(LIST UP TO 4 ANTIBIOTICS) (GO TO TABLE)***

|  | **Medicine 1** | **Medicine 2** | **Medicine 3** | **Medicine 4** |
| --- | --- | --- | --- | --- |
| Name of medicine |  |  |  |  |
| Medicine/package/bottle seen? (**Yes/No**) |  |  |  |  |
| - medicine seen and name legible (**Yes/No**) |  |  |  |  |
| - medicine seen and name illegible (**Yes/No**) |  |  |  |  |
| - Name available (**Yes/No**) |  |  |  |  |
| Was this medicine prescribed to you/them at a health facility? (**Yes/No**) |  |  |  |  |
| - **If YES**, at which health facility was it recommended/ prescribed? |  |  |  |  |
| - **If NO**, how did you know which medicine to buy? |  |  |  |  |
| From where did you/they get this medicine? |  |  |  |  |
| Did you/they buy/receive a full dose of the medication or just a part of a dose? (**Full/Part**) |  |  |  |  |
| For how many DAYS were you/they required to take the medicine? |  |  |  |  |
| Did you finish taking all the medicine? (**Yes/No**) |  |  |  |  |
| - **If NO**, for how many days did you take the medicine? |  |  |  |  |
| - **If NO**, what made you stop taking the medicine? |  |  |  |  |
| Did you/they change to a different medication or use any another medication when taking this one? (**Yes/No**) |  |  |  |  |
| - **If YES**, which other medication did you change to or use? |  |  |  |  |
| How did you/they know when you needed a different medicine from the one you were using? |  |  |  |  |
| Did any other person in this household use this same medicine? (**Yes/No**) |  |  |  |  |
