# Supplementary material for: Carriage of antimicrobial-resistant bacteria in a high-density informal settlement in Kenya is associated with environmental risk-factors
Source: Antimicrob Resist Infect Control. 2021 Jan 22;10:18. doi: 10.1186/s13756-021-00886-y (PMC7821723; doi:10.1186/s13756-021-00886-y)
Supplement: Supplementary file 3 — Additional file 3. Nine of the most abundant (found in > of all E. coli isolates) resistance phenotypes out of 148 unique combinations. The number of isolates and their proportional representation (%) of the total are shown. Profile marked with an asterisk (*) denotes the penta-resistant phenotype. [file 13756_2021_886_MOESM3_ESM.docx]

**Additional file 3: Nine of the most abundant (found in >10% of all *E. coli* isolates) resistance phenotypes out of 148 unique combinations.** The number of isolates and their proportional representation (%) of the total are shown. Profile marked with an asterisk (*) denotes the penta-resistant phenotype.

| **Unique profiles****^ŧ^** | **Stool (n = 23,981)** | **Hand swabs (n = 3,020)** | **Water (n = 2,354)** |
| --- | --- | --- | --- |
| AmpStrSulTetTmp* | 7,509 (31·3%) | 591 (19·6%) | 134 (5·7%) |
| SulTmp | 1,778 (7·4%) | 397 (13·1%) | 480 (20·4%) |
| AmpSulTmp | 1,263 (5·3%) | 276 (9·1%) | 281 (11·9%) |
| AmpStrSulTmp | 3,073 (12·8%) | 163 (5·4%) | 149 (6·3%) |
| AmpSulTetTmp | 1,291 (5·4%) | 213 (7·1%) | 201 (8·5%) |
| Tmp | 322 (1·3%) | 150 (5·0%) | 255 (10·8%) |
| Amp | 293 (1·2%) | 213 (7·1%) | 196 (8·3%) |
| SulTetTmp | 940 (3·9%) | 125 (4·1%) | 199 (8·5%) |
| AmpChlStrSulTetTmp | 1,808 (7·5%) | 164 (5·4%) | 15 (0·6%) |

^ŧ^Amp, ampicillin; Chl, chloramphenicol; Str, streptomycin; Sul, sulfamethoxazole; Tet, tetracycline; Tmp, trimethoprim. The proportion of multi-drug resistant isolates i.e. resistant to ≥3 antibiotic classes, was 85·6% for stool, 64·9% for hand swabs, and 49·7% for water samples.
