# Supplementary material for: Carriage of antimicrobial-resistant bacteria in a high-density informal settlement in Kenya is associated with environmental risk-factors
Source: Antimicrob Resist Infect Control. 2021 Jan 22;10:18. doi: 10.1186/s13756-021-00886-y (PMC7821723; doi:10.1186/s13756-021-00886-y)
Supplement: Supplementary file 4 — Additional file 4. Multivariable regression analysis for antimicrobial resistance load (Log10 CFU) at the household level. Only variables with P < 0·2 in the univariable mixed-effects model were included in the multivariable model. Regression estimates (β) and 95% confidence intervals with P < 0·05 are shown in bold. P = 0·00 indicates P < 0·01. [file 13756_2021_886_MOESM4_ESM.docx]

**Additional file 3: Multivariable regression analysis for antimicrobial resistance load (Log_10_ CFU) at the household level.** Only variables with *P* < 0·2 in the univariable mixed-effects model were included in the multivariable model. Regression estimates (β) and 95% confidence intervals with *P* < 0·05 are shown in bold. *P* = 0·00 indicates *P* < 0·01.

|  | **Ampicillin** | | **Streptomycin** | | **Sulfamethoxazole** | | **Tetracycline** | | **Trimethoprim** | |
| --- | --- | --- | --- | --- | --- | --- | --- | --- | --- | --- |
| **Variable** | β [95% CI] | *P* | β [95% CI] | *P* | β [95% CI] | *P* | β [95% CI] | *P* | β [95% CI] | *P* |
| Main water source*: |  |  |  |  |  |  |  |  |  |  |
| - *Public-protected* | -0·31 [-0·62, 0·00] | 0·05 | -0·16 [-0·51, 0·20] | 0·38 | -0·10 [-0·36, 0·16] | 0·44 | -0·26 [-0·63, 0·11] | 0·17 | -0·14 [-0·39, 0·10] | 0·25 |
| - *Private-unprotected* | 0·06 [-1·33, 1·45] | 0·93 | 0·30 [-1·27, 1·86] | 0·71 | 0·37 [-0·78, 1·51] | 0·53 | -0·29 [-1·92, 1·34] | 0·73 | 0·49 [-0·60, 1·58] | 0·38 |
| - *Public-unprotected* | -0·64 [-1·32, 0·04] | 0·07 | -0·06 [-0·83, 0·71] | 0·87 | -0·49 [-1·05, 0·08] | 0·09 | -0·64 [-1·45, 0·16] | 0·12 | -0·29 [-0·83, 0·25] | 0·29 |
| Time to fetch water (min) | 0·05 [-0·04, 0·14] | 0·26 | 0·03 [-0·07, 0·13] | 0·59 | 0·00 [-0·07, 0·08] | 0·91 | -0·01 [-0·12, 0·09] | 0·82 | 0·04 [-0·03, 0·11] | 0·27 |
| Days without water | 0·01 [-0·06, 0·07] | 0·82 | -0·01 [-0·08, 0·07] | 0·88 | -0·02 [-0·07, 0·03] | 0·40 | -0·02 [-0·10, 0·05] | 0·58 | -0·04 [-0·09, 0·01] | 0·12 |
| Treats water by boiling | 0·17 [-0·17, 0·52] | 0·33 | 0·16 [-0·23, 0·56] | 0·41 | 0·17 [-0·12, 0·46] | 0·25 | 0·20 [-0·21, 0·61] | 0·35 | **0·31 [0·04, 0·58]** | **0·03** |
| Last water treatment (day) | 0·02 [-0·11, 0·16] | 0·74 | -0·01 [-0·16, 0·15] | 0·94 | 0·02 [-0·09, 0·14] | 0·71 | -0·01 [-0·17, 0·15] | 0·88 | 0·05 [-0·06, 0·16] | 0·35 |
| Toilet cleaned | 0·32 [-0·20, 0·83] | 0·23 | -0·05 [-0·63, 0·54] | 0·88 | 0·38 [-0·05, 0·81] | 0·09 | 0·45 [-0·16, 1·06] | 0·15 | 0·37 [-0·04, 0·77] | 0·08 |
| Night toilet type: |  |  |  |  |  |  |  |  |  |  |
| - *Ventilator improved pit* | 0·46 [-0·34, 1·26] | 0·26 | 0·32 [-0·59, 1·23] | 0·49 | 0·07 [-0·59, 0·74] | 0·83 | -0·27 [-1·22, 0·67] | 0·57 | -0·06 [-0·69, 0·57] | 0·85 |
| - *Pit with slab* | 0·49 [0·00, 0·99] | 0·05 | 0·50 [-0·06, 1·07] | 0·08 | 0·13 [-0·28, 0·54] | 0·54 | -0·13 [-0·71, 0·46] | 0·67 | -0·04 [-0·43, 0·35] | 0·86 |
| - *Traditional* | 0·03 [-0·58, 0·63] | 0·93 | 0·25 [-0·44, 0·94] | 0·47 | -0·07 [-0·57, 0·43] | 0·79 | **-0·79 [-1·51, -0·08]** | **0·03** | -0·07 [-0·54, 0·40] | 0·77 |
| - *Bucket/plastic* | 0·26 [-0·23, 0·75] | 0·29 | 0·23 [-0·32, 0·79] | 0·41 | -0·06 [-0·47, 0·34] | 0·76 | **-0·62 [-1·19, -0·04]** | **0·04** | -0·24 [-0·62, 0·15] | 0·23 |
| - *No facilities/open field* | 0·46 [-0·29, 1·22] | 0·23 | 0·03 [-0·82, 0·88] | 0·95 | -0·02 [-0·64, 0·60] | 0·95 | -0·71 [-1·59, 0·18] | 0·12 | **-0·64 [-1·23, -0·04]** | **0·04** |
| HW with soap | **0·20 [0·01, 0·40]** | **0·04** | 0·21 [-0·01, 0·43] | 0·06 | 0·07 [-0·09, 0·23] | 0·37 | 0·19 [-0·03, 0·42] | 0·10 | 0·10 [-0·06, 0·25] | 0·22 |
| HW before feeding child | -0·06 [-0·18, 0·06] | 0·31 | -0·04 [-0·17, 0·09] | 0·58 | -0·08 [-0·18, 0·02] | 0·10 | -0·07 [-0·2, 0·07] | 0·34 | -0·06 [-0·15, 0·03] | 0·17 |
| HW facility location |  |  |  |  |  |  |  |  |  |  |
| - *Toilet within premises* | 0·14 [-0·15, 0·43] | 0·34 | 0·13 [-0·19, 0·46] | 0·42 | 0·10 [-0·13, 0·34] | 0·39 | 0·25 [-0·09, 0·59] | 0·15 | 0·17 [-0·05, 0·4] | 0·13 |
| - *Elsewhere on premises* | **0·47 [0·20, 0·73]** | **0·00** | 0·28 [-0·02, 0·58] | 0·07 | **0·35 [0·13, 0·57]** | **0·00** | **0·36 [0·05, 0·67]** | **0·02** | **0·22 [0·01, 0·43]** | **0·04** |
| - *No designated place* | **-0·28 [-0·55, -0·02]** | **0·04** | -0·13 [-0·44, 0·17] | 0·39 | 0·04 [-0·18, 0·26] | 0·72 | 0·26 [-0·05, 0·58] | 0·10 | -0·21 [-0·42, 0·00] | 0·05 |
| Enrolled child: |  |  |  |  |  |  |  |  |  |  |
| - *Time spent outside (h)* | 0·00 [-0·05, 0·05] | 0·98 | -0·01 [-0·08, 0·05] | 0·63 | 0·01 [-0·03, 0·06] | 0·52 | 0·03 [-0·03, 0·09] | 0·35 | 0·00 [-0·04, 0·04] | 0·97 |
| - *Eats soil* | **0·51 [0·26, 0·77]** | **0·00** | **0·39 [0·10, 0·68]** | **0·01** | **0·27 [0·06, 0·48]** | **0·01** | **0·54 [0·24, 0·84]** | **0·00** | **0·28 [0·08, 0·48]** | **0·01** |
| Rainfall (per mm) | **-2·56 [-3·60, -1·52]** | **0·00** | **-1·23 [-2·40, -0·06]** | **0·04** | -0·57 [-1·42, 0·28] | 0·19 | 1·18 [-0·03, 2·40] | 0·06 | **-1·19 [-2·00, -0·37]** | **0·00** |
| Father’s education level: |  |  |  |  |  |  |  |  |  |  |
| - *High school* | -0·02 [-0·35, 0·32] | 0·93 | 0·17 [-0·21, 0·56] | 0·38 | 0·10 [-0·18, 0·39] | 0·48 | 0·08 [-0·32, 0·48] | 0·71 | 0·03 [-0·23, 0·29] | 0·81 |
| - *College* | 0·96 [-0·22, 2·13] | 0·11 | 1·35 [-0·02, 2·72] | 0·05 | 0·72 [-0·29, 1·73] | 0·16 | 0·68 [-0·74, 2·1] | 0·35 | 0·47 [-0·45, 1·39] | 0·31 |
| Mother’s education level: |  |  |  |  |  |  |  |  |  |  |
| - *Primary school* | -0·52 [-1·17, 0·14] | 0·12 | -0·72 [-1·48, 0·05] | 0·07 | -0·25 [-0·82, 0·32] | 0·38 | -0·11 [-0·91, 0·68] | 0·78 | 0·02 [-0·50, 0·53] | 0·95 |
| - *High school* | **-0·87 [-1·58, -0·16]** | **0·02** | **-0·91 [-1·73, -0·08]** | **0·03** | -0·29 [-0·90, 0·32] | 0·35 | -0·26 [-1·11, 0·60] | 0·55 | 0·02 [-0·53, 0·57] | 0·95 |
| - *College* | -0·93 [-1·90, 0·05] | 0·06 | **-1·40 [-2·53, -0·26]** | **0·02** | -0·32 [-1·16, 0·52] | 0·46 | -0·70 [-1·88, 0·47] | 0·24 | 0·19 [-0·57, 0·95] | 0·62 |
| Altitude (10m increments) | 0·00 [-0·01, 0·00] | 0·24 | 0·00 [-0·01, 0·00] | 0·78 | 0·00 [-0·01, 0·00] | 0·05 | -0·01 [-0·01, 0·00] | 0·05 | 0·00 [-0·01, 0·00] | 0·06 |
| Household used antibiotic | 0·22 [-0·15, 0·58] | 0·24 | 0·19 [-0·22, 0·60] | 0·36 | 0·11 [-0·19, 0·41] | 0·48 | 0·33 [-0·09, 0·76] | 0·13 | 0·10 [-0·19, 0·39] | 0·50 |
| Respondent age (years) | **-0·02 [-0·03, -0·02]** | **0·00** | **-0·01 [-0·02, 0·00]** | **0·01** | 0·00 [-0·01, 0·00] | 0·20 | 0·00 [-0·01, 0·00] | 0·47 | **-0·01 [-0·01, 0·00]** | **0·02** |

*A protected source prevents contamination of water by the environment e.g. a source covered with a concrete slab or a completely covered tank; ^ŧ^Handwashing station located elsewhere within the household premises other than at a toilet facility or the household kitchen. HW: handwashing
