# Supplementary material for: Carriage of antimicrobial-resistant bacteria in a high-density informal settlement in Kenya is associated with environmental risk-factors
Source: Antimicrob Resist Infect Control. 2021 Jan 22;10:18. doi: 10.1186/s13756-021-00886-y (PMC7821723; doi:10.1186/s13756-021-00886-y)
Supplement: Supplementary file 5 — Additional file 5. Multivariable regression analysis for antimicrobial resistance load (Log10 CFU) at the adult level (≥18 years). Only variables with P < 0·2 in the univariable mixed-effects model were included in the multivariable model. Regression estimates (β) and 95% confidence intervals with P < 0·05 are shown in bold. P = 0·00 indicates P < 0·01. [file 13756_2021_886_MOESM5_ESM.docx]

**Additional file 3:** **Multivariable regression analysis for antimicrobial resistance load (Log_10_ CFU) at the adult level (≥18 years).** Only variables with *P* < 0·2 in the univariable mixed-effects model were included in the multivariable model. Regression estimates (β) and 95% confidence intervals with *P* < 0·05 are shown in bold. *P* = 0·00 indicates *P* < 0·01.

|  | **Ampicillin** | | **Streptomycin** | | **Sulfamethoxazole** | | **Tetracycline** | | **Trimethoprim** | |
| --- | --- | --- | --- | --- | --- | --- | --- | --- | --- | --- |
| **Variable** | β [95% CI] | *P* | β [95% CI] | *P* | β [95% CI] | *P* | β [95% CI] | *P* | β [95% CI] | *P* |
| Main water source*: |  |  |  |  |  |  |  |  |  |  |
| - *Public-protected* | **-0·47 [-0·89, -0·05]** | **0·03** | -0·30 [-0·75, 0·16] | 0·20 | -0·08 [-0·39, 0·22] | 0·59 | -0·19 [-0·65, 0·27] | 0·42 | -0·26 [-0·55, 0·03] | 0·07 |
| - *Private-unprotected* | 0·36 [-1·88, 2·60] | 0·75 | 0·49 [-1·88, 2·86] | 0·69 | 0·12 [-1·52, 1·76] | 0·89 | 0·83 [-1·57, 3·23] | 0·50 | 0·16 [-1·46, 1·78] | 0·85 |
| - *Public-unprotected* | -0·27 [-1·25, 0·70] | 0·58 | -0·09 [-1·12, 0·95] | 0·87 | -0·29 [-1·00, 0·42] | 0·43 | 0·24 [-0·80, 1·29] | 0·65 | -0·34 [-1·04, 0·35] | 0·33 |
| Days without water | 0·03 [-0·06, 0·13] | 0·50 | 0·01 [-0·09, 0·11] | 0·87 | -0·01 [-0·08, 0·06] | 0·83 | 0·03 [-0·07, 0·14] | 0·53 | -0·03 [-0·10, 0·04] | 0·42 |
| Toilet outside premises | 0·34 [-0·09, 0·77] | 0·12 | -0·08 [-0·54, 0·39] | 0·74 | 0·03 [-0·27, 0·34] | 0·82 | -0·33 [-0·80, 0·13] | 0·16 | 0·08 [-0·20, 0·36] | 0·59 |
| HW after urination | 0·07 [-0·08, 0·23] | 0·36 | -0·03 [-0·19, 0·14] | 0·75 | 0·04 [-0·08, 0·15] | 0·54 | **0·18 [0·01, 0·34]** | **0·04** | 0·04 [-0·07, 0·15] | 0·47 |
| HW before feeding child | -0·12 [-0·30, 0·06] | 0·21 | -0·05 [-0·24, 0·14] | 0·63 | -0·08 [-0·21, 0·05] | 0·21 | -0·06 [-0·25, 0·13] | 0·54 | -0·08 [-0·21, 0·04] | 0·20 |
| HW facility location: |  |  |  |  |  |  |  |  |  |  |
| - *Toilet within premises* | 0·23 [-0·18, 0·64] | 0·27 | 0·12 [-0·31, 0·56] | 0·58 | 0·05 [-0·24, 0·35] | 0·73 | 0·23 [-0·21, 0·67] | 0·31 | 0·11 [-0·17, 0·40] | 0·43 |
| - *Elsewhere on premises* | **0·51 [0·15, 0·88]** | **0·01** | 0·20 [-0·19, 0·59] | 0·32 | **0·30 [0·04, 0·57]** | **0·03** | **0·42 [0·02, 0·81]** | **0·04** | **0·29 [0·03, 0·54]** | **0·03** |
| - *No designated place* | **-0·48 [-0·85, -0·11]** | **0·01** | -0·24 [-0·64, 0·16] | 0·23 | -0·07 [-0·34, 0·20] | 0·60 | 0·23 [-0·17, 0·63] | 0·26 | -0·19 [-0·45, 0·06] | 0·13 |
| Child eats soil | **0·41 [0·02, 0·79]** | **0·04** | 0·24 [-0·17, 0·65] | 0·25 | **0·30 [0·02, 0·58]** | **0·04** | 0·32 [-0·09, 0·74] | 0·12 | 0·23 [-0·03, 0·50] | 0·09 |
| Children ≤ 5y (counts) | 0·28 [-0·08, 0·63] | 0·13 | 0·14 [-0·24, 0·53] | 0·46 | 0·20 [-0·05, 0·45] | 0·12 | -0·04 [-0·43, 0·34] | 0·83 | 0·14 [-0·10, 0·37] | 0·26 |
| Rainfall (per mm) | **-3·26 [-4·88, -1·65]** | **0·00** | -1·11 [-2·82, 0·59] | 0·20 | -0·65 [-1·84, 0·54] | 0·28 | 0·47 [-1·26, 2·20] | 0·59 | **-1·79 [-2·97, -0·61]** | **0·00** |
| School children (counts) | 0·03 [-0·11, 0·18] | 0·67 | -0·02 [-0·18, 0·14] | 0·80 | **-0·13 [-0·23, -0·03]** | **0·01** | -0·07 [-0·23, 0·09] | 0·37 | **-0·10 [-0·20, -0·01]** | **0·03** |
| Mother’s education level: |  |  |  |  |  |  |  |  |  |  |
| - *Primary school* | -0·74 [-1·69, 0·21] | 0·13 | -0·58 [-1·62, 0·46] | 0·28 | 0·07 [-0·59, 0·73] | 0·83 | -0·21 [-1·26, 0·82] | 0·69 | 0·26 [-0·33, 0·86] | 0·38 |
| - *High school* | **-1·17 [-2·15, -0·19]** | **0·02** | -0·76 [-1·84, 0·32] | 0·17 | -0·02 [-0·71, 0·66] | 0·94 | -0·44 [-1·51, 0·64] | 0·43 | 0·28 [-0·33, 0·89] | 0·37 |
| - *College* | **-1·78 [-3·09, -0·47]** | **0·01** | -1·42 [-2·85, 0·02] | 0·05 | -0·52 [-1·44, 0·39] | 0·26 | **-1·53 [-2·97, -0·10]** | **0·04** | 0·02 [-0·80, 0·84] | 0·96 |
| Adult age (years) | 0·00 [-0·03, 0·03] | 0·88 | 0·02 [-0·01, 0·05] | 0·15 | **0·03 [0·01, 0·05]** | **0·00** | 0·02 [-0·01, 0·05] | 0·13 | **0·03 [0·01, 0·05]** | **0·00** |

*A protected source prevents contamination of water by the environment e.g. a source covered with a concrete slab or a completely covered tank; ^ŧ^Handwashing station located elsewhere within the household premises other than at a toilet facility or the household kitchen. HW: handwashing.
