# Supplementary material for: Carriage of antimicrobial-resistant bacteria in a high-density informal settlement in Kenya is associated with environmental risk-factors
Source: Antimicrob Resist Infect Control. 2021 Jan 22;10:18. doi: 10.1186/s13756-021-00886-y (PMC7821723; doi:10.1186/s13756-021-00886-y)
Supplement: Supplementary file 6 — Additional file 6. Multivariable regression analysis for antimicrobial resistance load (Log10 CFU) for children aged 0–5 years. Only variables with P < 0·2 in the univariable mixed-effects model were included in the multivariable model. Regression estimates (β) and 95% confidence intervals with P < 0·05 are shown in bold. P = 0·00 indicates P < 0·01. [file 13756_2021_886_MOESM6_ESM.docx]

**Additional file 4:** **Multivariable regression analysis for antimicrobial resistance load (Log_10_ CFU) for children aged 0-5 years.** Only variables with *P* < 0·2 in the univariable mixed-effects model were included in the multivariable model. Regression estimates (β) and 95% confidence intervals with *P* < 0·05 are shown in bold. *P* = 0·00 indicates *P* < 0·01.

|  | **Ampicillin** | | **Streptomycin** | | **Sulfamethoxazole** | | **Tetracycline** | | **Trimethoprim** | |
| --- | --- | --- | --- | --- | --- | --- | --- | --- | --- | --- |
| **Variable** | β [95% CI] | *P* | β [95% CI] | *P* | β [95% CI] | *P* | β [95% CI] | P | β [95% CI] | P |
| Main water source*: |  |  |  |  |  |  |  |  |  |  |
| - *Public-protected* | -0·25 [-0·60, 0·11] | 0·17 | -0·10 [-0·53, 0·34] | 0·67 | -0·14 [-0·47, 0·19] | 0·41 | -0·42 [-0·86, 0·02] | 0·06 | -0·06 [-0·37, 0·24] | 0·68 |
| - *Private-unprotected* | -0·35 [-2·00, 1·30] | 0·68 | 0·12 [-1·90, 2·14] | 0·91 | 0·56 [-0·97, 2·09] | 0·47 | -1·22 [-3·38, 0·95] | 0·27 | 0·76 [-0·67, 2·18] | 0·30 |
| - *Public-unprotected* | -0·69 [-1·55, 0·17] | 0·12 | 0·08 [-0·97, 1·13] | 0·88 | -0·30 [-1·10, 0·49] | 0·46 | -0·83 [-1·95, 0·29] | 0·15 | 0·00 [-0·74, 0·74] | 1·00 |
| Treats water by boiling | 0·10 [-0·34, 0·53] | 0·66 | 0·04 [-0·48, 0·57] | 0·87 | 0·09 [-0·31, 0·49] | 0·66 | -0·04 [-0·58, 0·51] | 0·89 | 0·22 [-0·15, 0·59] | 0·25 |
| Last water treatment (days) | -0·08 [-0·24, 0·09] | 0·36 | -0·03 [-0·24, 0·17] | 0·74 | -0·04 [-0·19, 0·11] | 0·62 | -0·02 [-0·23, 0·19] | 0·85 | -0·05 [-0·20, 0·09] | 0·45 |
| Toilet cleaned | 0·50 [-0·15, 1·15] | 0·13 | 0·21 [-0·58, 1·00] | 0·61 | 0·47 [-0·14, 1·07] | 0·13 | 0·90 [0·09, 1·72] | 0·03 | 0·36 [-0·20, 0·91] | 0·20 |
| Night toilet type: |  |  |  |  |  |  |  |  |  |  |
| - *Ventilator improved pit* | 0·03 [-0·95, 1·00] | 0·95 | 0·46 [-0·73, 1·64] | 0·45 | 0·25 [-0·65, 1·16] | 0·59 | -0·59 [-1·81, 0·64] | 0·35 | 0·02 [-0·81, 0·86] | 0·96 |
| - *Pit with slab* | 0·24 [-0·38, 0·86] | 0·44 | 0·41 [-0·35, 1·17] | 0·29 | 0·18 [-0·39, 0·76] | 0·54 | -0·38 [-1·17, 0·41] | 0·35 | 0·06 [-0·48, 0·59] | 0·84 |
| - *Traditional* | 0·00 [-0·75, 0·75] | 1·00 | 0·25 [-0·66, 1·16] | 0·59 | 0·14 [-0·56, 0·84] | 0·70 | -1·39 [-2·33, -0·45] | 0·00 | 0·20 [-0·44, 0·85] | 0·53 |
| - *Bucket/plastic* | -0·08 [-0·69, 0·53] | 0·79 | 0·19 [-0·55, 0·93] | 0·62 | -0·01 [-0·57, 0·56] | 0·98 | -0·99 [-1·77, -0·21] | 0·01 | -0·17 [-0·70, 0·35] | 0·52 |
| - *No facilities/open field* | -0·07 [-1·05, 0·91] | 0·89 | -0·43 [-1·63, 0·76] | 0·48 | -0·19 [-1·09, 0·72] | 0·69 | -1·28 [-2·56, 0·00] | 0·05 | -0·78 [-1·63, 0·06] | 0·07 |
| HW after urination | **0·20 [0·03, 0·38]** | **0·02** | 0·06 [-0·16, 0·27] | 0·59 | 0·12 [-0·04, 0·28] | 0·15 | 0·08 [-0·14, 0·31] | 0·47 | 0·19 [0·04, 0·34] | 0·01 |
| HW w/ soap after urination | -0·11 [-0·45, 0·23] | 0·52 | 0·13 [-0·28, 0·55] | 0·53 | -0·04 [-0·35, 0·28] | 0·81 | -0·14 [-0·58, 0·31] | 0·55 | -0·13 [-0·43, 0·16] | 0·37 |
| HW after defecation | 0·86 [-0·29, 2·01] | 0·14 | 0·75 [-0·66, 2·16] | 0·29 | 0·63 [-0·43, 1·70] | 0·24 | 0·32 [-1·18, 1·83] | 0·67 | 0·62 [-0·37, 1·62] | 0·22 |
| HW facility location: |  |  |  |  |  |  |  |  |  |  |
| - *Toilet within premises* | 0·12 [-0·23, 0·47] | 0·50 | 0·19 [-0·24, 0·62] | 0·39 | 0·17 [-0·16, 0·50] | 0·32 | 0·28 [-0·18, 0·74] | 0·23 | 0·28 [-0·03, 0·58] | 0·08 |
| - *Elsewhere on premises* | **0·45 [0·12, 0·78]** | **0·01** | 0·39 [-0·01, 0·79] | 0·06 | **0·35 [0·05, 0·66]** | 0·02 | 0·26 [-0·16, 0·68] | 0·23 | 0·28 [0·00, 0·57] | 0·05 |
| Child eats soil | **0·59 [0·27, 0·91]** | **0·00** | 0·52 [0·14, 0·91] | 0·01 | 0·22 [-0·08, 0·52] | 0·14 | 0·80 [0·40, 1·21] | 0·00 | 0·25 [-0·03, 0·52] | 0·08 |
| Rainfall (per mm) | **-2·02 [-3·28, -0·76]** | **0·00** | -1·26 [-2·80, 0·27] | 0·11 | -0·69 [-1·85, 0·47] | 0·25 | 1·74 [0·07, 3·40] | 0·04 | -0·82 [-1·91, 0·27] | 0·14 |
| Father’s education level: |  |  |  |  |  |  |  |  |  |  |
| - *High school* | -0·19 [-0·56, 0·17] | 0·30 | 0·14 [-0·30, 0·58] | 0·54 | 0·05 [-0·29, 0·39] | 0·76 | 0·06 [-0·36, 0·48] | 0·79 | -0·04 [-0·35, 0·26] | 0·78 |
| - *College* | 1·07 [-0·28, 2·41] | 0·12 | 1·43 [-0·19, 3·05] | 0·08 | 1·10 [-0·15, 2·36] | 0·09 | 1·38 [-0·21, 2·98] | 0·09 | 1·09 [-0·04, 2·23] | 0·06 |
| Altitude (10m increments) | **-0·01 [-0·01, 0·00]** | **0·03** | 0·00 [-0·01, 0·01] | 0·85 | **-0·01 [-0·01, 0·00]** | 0·04 | -0·01 [-0·01, 0·00] | 0·09 | -0·01 [-0·01, 0·00] | 0·00 |
| Household used antibiotic | 0·39 [-0·07, 0·84] | 0·10 | 0·22 [-0·34, 0·77] | 0·45 | **0·46 [0·04, 0·88]** | 0·03 | 0·10 [-0·49, 0·69] | 0·74 | 0·42 [0·02, 0·81] | 0·04 |
| Respondent age (years) | -0·02 [-0·17, 0·13] | 0·79 | -0·11 [-0·29, 0·07] | 0·22 | -0·08 [-0·22, 0·06] | 0·25 | -0·06 [-0·23, 0·11] | 0·49 | -0·10 [-0·22, 0·02] | 0·11 |

*A protected source prevents contamination of water by the environment e.g. a source covered with a concrete slab or a completely covered tank; ^ŧ^Handwashing station located elsewhere within the household premises other than at a toilet facility or the household kitchen. HW: handwashing
